# Editorial: Using methods and approaches from behavioral ecology to address issues in applied animal sciences

**DOI:** 10.3389/fvets.2025.1622183

**Published:** 2025-06-10

**Authors:** Edward A. Codling, Simon P. Turner, Victoria Lee, Gareth Arnott

**Affiliations:** ^1^School of Mathematics, Statistics and Actuarial Science, University of Essex, Colchester, United Kingdom; ^2^Animal and Veterinary Sciences Department, Scotland's Rural College (SRUC), Edinburgh, United Kingdom; ^3^Institute for Global Food Security, School of Biological Sciences, Queen's University Belfast, Belfast, United Kingdom

**Keywords:** behavioral ecology, applied animal sciences, animal welfare, movement ecology, animal behavior

Behavioral ecology is the study of animal behavior in an ecological context. Specific areas of focused research include both intra- and inter-species interactions such as methods of communication and signaling, predation and avoidance, conflict and competition, cooperation and altruism, mating systems and parental care, and a range of other direct and indirect social interactions that may be considered in an evolutionary context. Behavioral interactions can also relate to the physical landscape animals inhabit, including habitat choice, resource use, territory, home range, and, more generally, the movement and space-use of animals at different scales and in different environments (a recently developing sub-field known as movement ecology).

Research in the field of behavioral ecology has dramatically increased over the last 20 years, mainly due to the improved accuracy and reduced cost of reliable trackers, loggers, sensors, and cameras for recording the behavior of animals at high temporal resolution for long periods of time. In parallel, new mathematical, statistical and computational methods have been developed to model, analyze and interpret these increasingly rich (and large) behavioral data sets. However, much of the focus of these new technologies and methods has been in the context of studying wild animals; there has been relatively little crossover of these new approaches into the field of applied animal sciences, where animals may be directly managed and/or inhabit (relatively) controlled environments. The goal of this interdisciplinary Research Topic is to explore opportunities for ideas, approaches, methods and techniques developed within the broad field of behavioral ecology to be adapted and used to address applied animal science issues, including improved animal management and welfare.

In the first manuscript in this Research Topic, Lee et al. review how fundamental theory relating to social behavior can improve farm animal welfare. The authors start by discussing the importance and relevance of using principles from behavioral ecology to understand the social behavior of farm animals, outlining that domesticated species retain an “evolutionary legacy” that influences their social behavior. Given the dynamic and challenging social conditions experienced on farms this has important implications for welfare. The review explores how an understanding of agonistic behavior informed by game theory can be used to improve farm animal welfare, with a subsequent section outlining the benefits related to positive social interactions. Importantly, the review concludes by highlighting a range of future research opportunities through the lens of Tinbergen's questions and illustrating the scope for collaboration between fundamental and applied ethologists.

The second contribution by Prentice et al. offers new perspectives on how animal personality could be exploited to reduce chronic stress in captive fish populations. The authors start by noting how, in a simplistic sense and in the longer term, welfare of captive fish populations could be improved by using selective breeding to improve the fit of the fish to their captive environment. They subsequently present a hypothetical argument that better understanding of “animal personality,” a concept widely discussed in behavioral ecology, could help to inform selective breeding strategies aimed at improving welfare outcomes for captive fish. Their basic premise is that since many “personality” traits are known to have a genetic basis then measurable (behavioral) stress responses at the individual level could act as useful biomarkers. The authors provide an overview of what properties a hypothetical behavioral biomarker should have in order to help inform selective breeding and hence improve chronic stress resistance in captive fish. Finally, they finish by outlining what further work would be needed to test and develop this idea so it could become a reality.

The third manuscript by Jowett et al. demonstrates how social network analysis methods can be used to highlight preferential associations in a dynamic sow herd. The article begins by outlining the fitness and welfare enhancing benefits of preferential affiliative associations between individuals, and how such associations have been rarely studied in livestock. The article studies preferential relationships in a herd of sows where the group composition changes frequently. Associations were studied by applying social network analysis to quantify the centrality of individuals in their group and the extent to which assortment was dictated by traits such as parity. Use of social network analysis to study the behavior of captive animals is still in its infancy and this article introduces informative new methods, especially the use of brokerage typologies to understand the role of individuals in the flow of interactions between subgroups of individuals in the group. The article demonstrates that preferential associations exist but do not require reciprocity. The existence of preferential associations raises important questions about the welfare implications of breaking these ties when group composition is changed for management purposes.

The fourth article by Chopra, Enticott, et al. reports on a pilot study where commercially available dog tracking Global Positioning System (GPS) sensors are used to explore the movement ecology of farm dogs. In movement ecology, animal home ranges are often estimated from GPS tracking data in order to better understand behaviors such as foraging, territoriality, and more general population dynamics. Meanwhile, farm dogs are a key working animal on most farms but have previously been rarely studied using these types of tracking approaches. The authors report and analyze the movements of three farm dogs tracked over 2 weeks and highlight different movement and space-use patterns, with notably distinct variation in locations visited between individuals. Although this study has a small sample size, there are nonetheless interesting findings that could be investigated in future studies, such as the fact that farm dogs were observed to spend more time near field gates and boundaries, where potential interactions with livestock and wildlife may take place.

In the fifth and final contribution to the Research Topic, Chopra, Cameron, et al. demonstrate how the grazing activity of cattle can be mapped and analyzed using commercial virtual fencing technology. Virtual fencing technology uses animal-mounted GPS tracking sensors to monitor and restrict individual animals within a user-controlled virtual fence. Interestingly, most commercial virtual fencing systems also automatically and continuously collect a wide range of other useful data, such as animal activity and temperature. In this study of three cattle grazing over 8 weeks in a UK pasture with a standard commercial virtual fencing system, the authors highlight how activity measured by the animal-mounted sensors strongly correlate with direct *in situ* observations of cattle grazing. They subsequently demonstrate how this sensor activity data can be combined with GPS location data so that grazing activity can be mapped spatiotemporally, providing additional insights into behavior and potentially helping with livestock management. The authors also highlight how measurements made by the animal-mounted sensors can be used to map temperature variations within the pasture.

This Research Topic has highlighted how methods and approaches originally developed in the field of behavioral ecology can be adapted and used to help inform ongoing research within applied animal sciences. The ideas, approaches, and case studies presented in this Research Topic act as important examples of the value gained by sharing knowledge and working across these disciplines.

